# Developing the Agile Institute, an effort to incorporate agile methodologies into Hackensack Meridian Health

**DOI:** 10.3389/fpubh.2025.1661374

**Published:** 2025-10-30

**Authors:** Lama El Zein, Victor A. Carrillo, Robert Bayly, Erin Glantz, Bethany Gregg, Mary Grove, Jose Azar

**Affiliations:** ^1^Hackensack Meridian School of Medicine, Nutley, NJ, United States; ^2^Hackensack Meridian Health, Edison, NJ, United States

**Keywords:** implementation science, quality improvement, Agile Science, organizational development, sprints

## Abstract

The persistent challenge of implementing meaningful and sustainable change in healthcare is well-documented. Barriers include resource limitations, technical insufficiencies, and resistance from entrenched processes and systems within hospitals, clinics, and health systems. Traditional quality improvement (QI) frameworks, while valuable, often fall short in addressing the variability and unpredictability of human behavior and decision-making that reflects the uniqueness of individual experiences and backgrounds working together in a complex organization. In response, Hackensack Meridian Health (HMH), a large integrated health system, established the Agile Institute to promote and diffuse methodologies from Agile Science (sprints, feedback loops, techniques from behavioral psychology to encourage certain behaviors, etc.) as a means to accelerate and sustain quality improvement efforts in care and patient outcomes. This narrative case study describes the conception, structure, and impact of the Agile Institute at HMH. The Institute was designed around three core pillars: training and education, consultation, and organizational identity development. Bootcamps and certification programs equipped staff across the health system with the knowledge and mindset needed to apply Agile. Consultative groups facilitated co-design sessions and iterative sprints, fostering collaboration and interdisciplinary development and implementation of innovative solutions. Intentional brand development helped to build engagement and credibility in both internal and external audiences. Over its first year, the Agile Institute achieved significant milestones: training over 130 staff, launching collaborative physician networks, and supporting system-wide initiatives that improved standardization and patient outcomes. The Institute’s approach-grounded in psychological safety, stakeholder co-design, and iterative feedback-demonstrated the value of embedding Agile principles not only in QI projects but also in organizational culture. Lessons learned highlight the importance of a minimally viable, adaptable structure and the necessity of aligning Agile strategies with both system and individual priorities. The HMH Agile Institute offers a replicable model for other healthcare organizations seeking to drive sustainable, system-wide transformation through Agile.

## 1 Introduction

A primary challenge for many healthcare delivery systems is implementing sustainable change in everyday practice. There can be significant lag time from “discovery to delivery” (1–4) due to a variety of factors that impede organizations’ ability to implement changes. These factors may include practical limitations related to funding, resource availability, or technical insufficiency (5, 6), and cultural or structural barriers (5–7).

Lack of clinician engagement can also inhibit QI efforts, and may be attributed to a variety of causes including inadequate knowledge or training in QI (8–11), and opinions that time and resources required to implement change may be better spent elsewhere (8, 10) or that QI is not their responsibility (8, 12).

In addition to organizational characteristics and the level of clinician engagement, the process used to implement QI can also influence the sustainability of quality interventions. QI efforts are more effective when they are implemented through collaboration, shared decision making between stakeholders, and the use of evaluation and feedback (13). These signal that in addition to the structural and cultural characteristics of a healthcare system, components related to how change is planned, developed, tested, and implemented can also influence the likelihood that it is adopted and sustained. Traditional tools for QI like Plan-Do-Study-Act (PDSA), Lean, and Six Sigma can be effective, but when applied, poor adherence to these frameworks’ key features along with weak implementation designs can doom QI initiatives (14–16). Traditional frameworks also tend to focus on processes and systems without specifically addressing human nature and factors that influence decision-making and behavior choices within the complex nature of healthcare organizations (16, 17). There is growing research indicating that healthcare organizations are complex adaptive systems (18–21), highlighting a need for QI frameworks that acknowledge that structure.

One of the emerging frameworks for creating real and sustainable change in healthcare is that of Agile. In general, Agile reflects the idea of using short, iterative sprints to foster quick learnings and continuous adjustments to facilitate change. Implementation scientists have attempted to build on common Agile principles through specific frameworks for the development and implementation of evidence-based practices (22, 23). While there are other evidence-based quality improvement (EBQI) frameworks that seek to incorporate evidence-based interventions into QI efforts through iteration and a collaborative process (24, 25), Agile methodologies stress speed and flexibility to foster rapid improvement. Some Agile frameworks also leverage concepts and theories from behavioral psychology, network science, and complexity science to foster the adoption and long-term sustainability of Agile-implemented solutions. Several of these frameworks were developed and refined at the Indiana University Center for Health Innovation and Implementation Science (CHIIS) (26). When taken together, the concepts, tools, and methodologies have been referred to as Agile Science (27–29).

Methodologies from Agile Science facilitate faster diffusion of successful interventions by identifying minimally viable components of those interventions so that they can be tailored to meet the needs of other organizations, facilities, or systems. This allows stakeholders to maintain their autonomy by “locally” operationalizing the solution based on their specific resources and situation, including incorporating specific evaluation and termination criteria to help ensure either rapid adoption or termination of ineffective solutions. Even before its refinement into methodologies within Agile Science, Agile has long been considered relevant for the ongoing digital transformation across the healthcare landscape. Previous literature examined the use of Agile in digital health software (30, 31), where it was deemed effective but underutilized. Agile has also been incorporated into a proposed framework for researching and evaluating mobile health technology (32), while others have suggested applying Agile concepts to improve care practices, bolster efficiency, manage risk, and engage patients (33, 34). In each case, components of Agile are lauded as fitting well with the nature of healthcare delivery, which often requires flexibility, rapid cycles of innovation, and regular feedback from patients and clinicians to drive change and growth.

The addition of concepts from network science and other disciplines compound the effectiveness of traditional Agile methods by accelerating the rate at which innovations are spread and adopted across organizations and systems. By using network mapping to identify persons with influence, frameworks from Agile Science encourage clinician engagement, continuously assess demand, and intentionally select stakeholders and messengers. Using specific Agile Science frameworks, hospitals and health systems have successfully evoked sustainable change in a variety of settings and situations. Examples include reductions in central-line infections in the intensive care unit (35), increased enrollment in a “hospital at home” program (36), and the adoption and implementation of a new dementia care model (18). More information on Agile Science and related frameworks, such as Agile Implementation (an eight-step process for implementing evidence-based solutions into everyday practice) and Agile Innovation (a process for finding and testing novel solutions that can be implemented), is available from a variety of resources (22, 23, 28, 37).

Much of the information on Agile and its role in improving the efficiency and delivery of healthcare is disparate, requiring healthcare professionals to hunt for relevant tools and identify a variety of sources to meet their needs. If consolidated into a single institute, the concepts, techniques, and applications of Agile and related frameworks could be taught and disseminated more efficiently to those who would benefit from their use. The current paper describes the experience of creating an Agile Institute at Hackensack Meridian Health (HMH), a large health system comprising 18 hospitals and more than 36,000 employees, to facilitate the learning of Agile and diffuse the use of Agile tools for improving the process and outcomes of care delivery.

## 2 Context

Like many health systems, HMH became aware of the barriers and challenges to implementing meaningful and sustainable improvements across the system several years ago and at that time sought a methodology that would enable rapid and adaptive improvements for various initiatives. Starting in 2022, the system began applying frameworks from Agile Science like Agile Implementation and Agile Innovation to various initiatives, including those intended to enhance performance in national rankings, align individual hospitals’ goals with network strategies, and improve collaboration between physicians and the administration.

The success of these initial efforts fostered a desire to spread the information regarding Agile throughout the health system and created a need to expand the capabilities of organization members to more effectively integrate these methodologies into their work. The approach adopted was to certify a few leaders in key positions across the network through a one-year certificate program at Indiana University through the CHIIS (38). This specialized training would create certified Agile Change Conductors who would share their knowledge with others in the organization and who would create Agile tools to be used across the organization.

It became clear that incorporating Agile across HMH required more than could be achieved from ad-hoc training sessions and distributing some reading material. Given the size and scope of the health system, a more formal structure was required to ensure consistency in the training and efficiency in the transfer of knowledge and skill sets. The creation of an Agile Institute was deemed to be the best option to formalize this process and diffuse and accelerate the use of Agile throughout the organization more broadly. The goal in creating the Agile Institute was to increase leadership buy-in and clinician engagement in QI efforts, train clinicians and staff in techniques to implement and foster sustainable change, further the quality improvement priorities of the health system, and build formal and informal peer-to-peer interactions. This would allow the system as a whole to apply needed changes to improve patient outcomes and care delivery, whether those changes involved processes, clinician practice, organizational efficiency, or others. The Institute would also provide a vehicle to consult with outside organizations who wanted assistance learning and applying Agile.

## 3 Key programmatic elements

### 3.1 Initial conception and creation

Five individuals, all of whom reported to the network’s Chief Quality Officer (CQO), enrolled in the one-year certificate program at Indiana University. While they were enrolled, the CQO enlisted those five individuals to develop a plan for the creation of the Agile Institute at HMH using knowledge gained from the certificate program. They determined that the most effective way to develop a plan was to approach the Agile Institute as if it was a startup company, with each member of the team serving in a specific role at that startup (e.g., Chief Executive Officer, Chief Financial Officer, etc.). The team then applied concepts they learned in the certificate program to develop a minimally viable plan for the creation of the Institute. They specified minimally viable goals for the first year that included: secure engagement from clinicians and administrators who are decision-makers regarding the allocation of time and resources to the creation of the Institute, develop the overall structure and specific components of the Institute within a year and identify the initial capital requirements (e.g., time and space), incorporate timely feedback loops (another Agile concept) to gather information from clinicians and staff across the system regarding interest and preferences related to the structure and services provided by the Institute, and create psychological safety to encourage openness about failures and successes. Through this process, the team successfully created the following structure and components.

### 3.2 Institute structure

**Figure 1:**
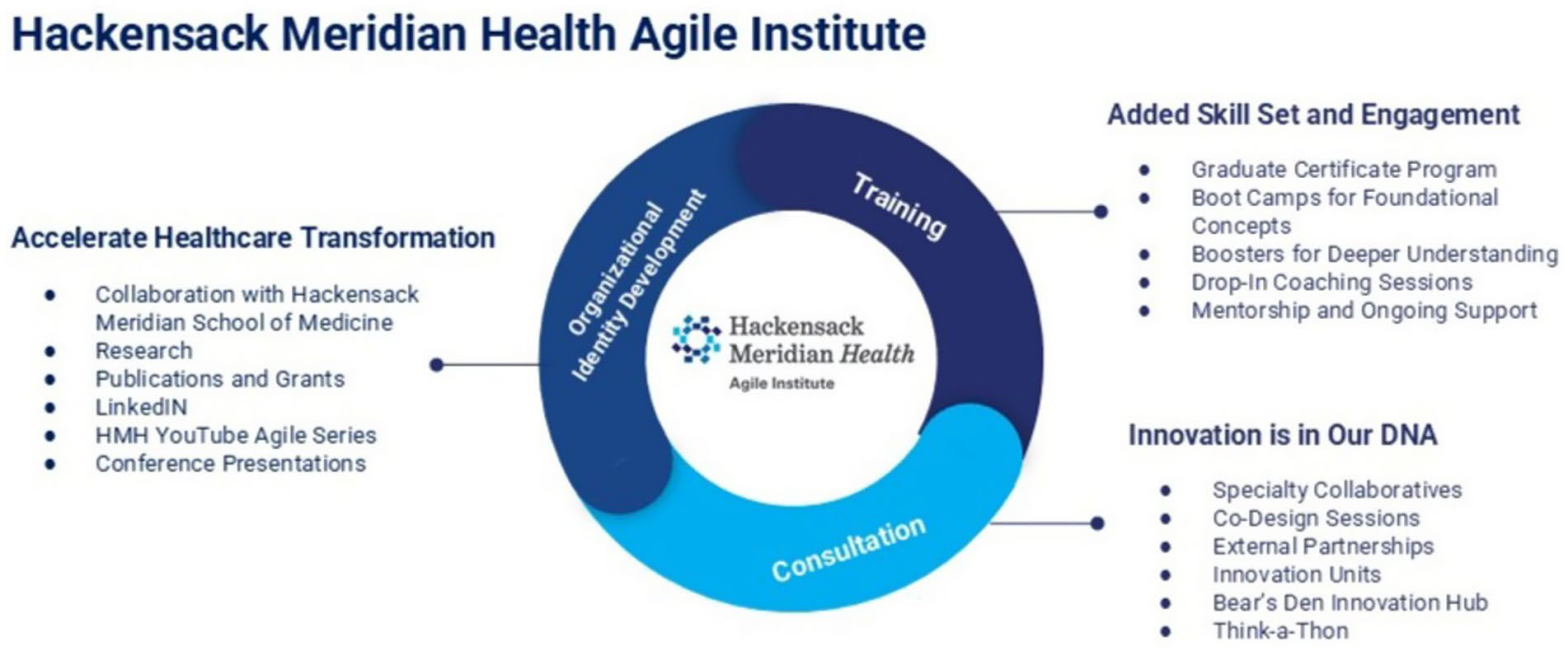
The structure of the Agile Institute. The Hackensack Meridian Health Agile Institute is built on three interconnected pillars—Training, Consultation, and Organizational Identity Development—to foster an agile mindset and culture. Training equips staff through certificate programs, boot camps, coaching, and mentorship. Consultation supports partners through co-design sprints, innovation units, and collaborations with the Bear’s Den, an internal innovation hub where teams and external partners co-develop and pitch new ideas. Organizational identity development drives transformation through research, publications, social media, and presentations, promoting rapid, sustainable quality improvement.

It was agreed early on that the Agile Institute would consist of three main components that are interrelated and that foster an agile mindset and culture across the health system (Figure 1). These three components are:

- *Training/coaching:* aimed at individual clinicians and staff throughout the health system, this included various programs, educational seminars, and materials for teaching Agile, as well as mentoring to provide on-going support; these are provided by those certified as Agile Change Conductors by completing the one-year graduate certificate program from the Indiana University CHIIS.
- *Consultation:* for internal departments at any of the system’s hospitals as well as outside organizations needing assistance with conducting QI initiatives, this involved intentional collaborations and co-design internal to the health system as well as partnerships and engagements with external entities to promote QI at other organizations and facilities; these were provided by the core team including the Agile Change Conductors and others these individuals had previously trained in the Agile Science methodologies.
- *Organizational identity development (image building):* this required performing research on the use of Agile as well as disseminating information regarding these methodologies and their effectiveness through publications, social media, YouTube, and conference presentations. These activities were carried out by those initially involved in the creation of the Institute as well as some administrative assistants added along the way.

### 3.3 Training and coaching

The goal of training and education is to equip clinicians and staff across the health system with the mindset and approach to thrive in the increasingly complex and unpredictable environment within which they work every day. The Institute incorporates three types of training and education: bootcamps, the Indiana University graduate certificate program, and one-on-one coaching/mentorships.

Agile Bootcamps were originally 2-day summits (about 6 h each day). However, this cadence was adjusted based on feedback from early participants, and currently the bootcamp schedules are tailored to the needs of specific groups who enroll. During the bootcamps, participants spend time immersed in the methodologies of Agile Implementation and Agile Innovation. Key topics of Agile are presented by faculty alongside case studies and group activities to allow participants to internalize the material. Step-by-step methodologies for discovering and implementing evidence-based practices as well as health innovations are taught and cover many Agile principles, including the agile mindset, storytelling, confirming demand, iterative sprints, and others. Participants also learn how to apply the Transformation Cycle to drive continuous improvement and how to run an Innovation Forum at their own facility to build engagement and promote meaningful change. Importantly, the Agile Bootcamp content and structure can be tailored to the specific needs of external audiences. This ensures that the material is relevant regardless of whether audiences represent large health systems, professional service firms, academic institutions, or some other type of organization.

The bootcamps also served to help secure support from health system executives for the Agile Institute. Representatives from several departments across the health system heard about the success others had achieved using Agile and reached out to the CQO and the Agile Institute team to learn more. Members of many of those departments enrolled in bootcamps and served as some of the first consultation clients. As demand grew, the team developing the Institute created “office hours” during which health system members could attend or call with questions regarding situations they encountered when attempting to leverage Agile. The interest in the bootcamps and office hours signaled to health system executives that demand existed for a broader and more formalized process to disseminate Agile, thereby ensuring demand for the Institute. Over time, the use of office hours waned, and these sessions were eventually discontinued based on a pre-determined termination plan, replaced by focused follow-up with interested bootcamp attendees.

One-on-one coaching and mentorship is provided by the Agile Change Conductors. These services are offered to any clinician or staff who requests it. Those requesting these services are familiar with the Institute or attended other events such as the bootcamps. They recognize the benefits the Institute can provide and reach out for assistance with an initiative they are working on. Other times, opportunities for coaching and mentorship are identified at the executive leadership level, where it is thought that these services would be beneficial for an individual or group struggling with a quality improvement issue.

Mentors are selected by the Agile Strategic committee, a group of individuals within the Agile Institute trained in Agile Methodologies and familiar with the health system. Once a mentor or coach is selected, meetings occur at least monthly, although they can be more frequent if requested by the mentee or those being coached. During these meetings, the individual or team working to improve care present their progress with their current quality improvement initiative and discuss any barriers or challenges they are facing. The coach or mentor will guide them through the components of the Agile Science methodologies to help apply the methodologies to the initiative. For example, they may discuss where demand for the solution exists, help map the network of individuals involved in the proposed improvement, or review the evaluation and termination plans.

HMH has entered into an informal agreement with Indiana University regarding the graduate certificate program. Those who are identified and/or interested in pursuing a Graduate Certificate in Innovation and Implementation Science from the Indiana University School of Medicine will complete six courses that cover implementation science, outcomes and evaluation, and leading of teams and projects. The certificate program provides a solid foundation for how to enhance care processes, influence patient and clinician behaviors, and drive effective and sustainable change by learning to apply Agile. Thus far, graduates have included physicians, nurses, consultants, administrators, executives, and researchers, and have come from various backgrounds and types of healthcare delivery systems. Recognizing the value of this educational pathway, the HMH School of Medicine is actively working in collaboration with leadership at the Agile Institute to develop a more formalized post-baccalaureate program that aligns with and expands upon these core competencies, reinforcing a long-term commitment to building agile change conductors.

### 3.4 Consultation

Consultation by members of the Agile Institute includes both internal and external audiences. For employees and clinicians within the health system, the Agile Institute consults on determining QI needs, designing interventions, and deploying sprints to facilitate change. An example of a common consultative activity would be to hold a “co-design sprint,” which is a rapid, iterative exercise where all those present participate and collaborate to explore ideas and possible solutions to an identified need. It involves the Institute team facilitating the development of specific and targeted interventions or minimal viable/essential specifications for an identified need. Co-design is a key aspect of Agile (22) and reflects a situation where all team members ideate together to create the initial version of QI efforts and determine how to define and measure success. Developing interventions in this way leverages strengths and knowledge of multiple disciplines and perspectives, increasing the likelihood that an intervention will be feasible and effective.

Another example of how the Institute provides consultation is by facilitating implementation sprints, which operationalize co-designed solutions through the Agile Transformation Cycle. These sprints identify early adopters (i.e., those most likely to accept and implement an innovative solution), remove barriers to adoption, and iterate based on real-time feedback from end users. This process accelerates progress while ensuring alignment with frontline realities and system priorities. Regardless of how the Agile Institute provides consultation, their services are grounded in collaboration, responsiveness, and a deep understanding of change management in complex health environments. Two examples of consultative activities include the creation of high-reliability units and the use of Innovation Units, described below.

#### 3.4.1 Creating high-reliability units

HMH strives to provide safe, high-quality, meaningful patient experiences through the consistent integration of high-reliability behaviors (i.e., consistently and repeatedly following best practices known to result in safe and high-quality care for patients) at the unit level. To further this goal, the Agile Institute was asked to consult by facilitating co-design sessions with key stakeholders from multiple hospital teams to assist in the creation of high-reliability units. These stakeholders can include clinicians, staff, and administrators from the hospitals and individual units. Representatives from Infection Prevention, Risk, Quality, Safety, and Patient Experience were all included in the co-design sessions. The initial session allowed these teams to brainstorm components they felt were key to promote safe, high-quality, and meaningful experiences. The representatives who attended the consultative activity defined the following core essentials:

1. A shared vision for “teamness” built on a strong foundation of psychological safety.
2. Dedicated time, space for all departments and teams to meet, round, and have a presence together with leadership support/administrative assistance as needed.
3. A method and means to “close the loop” and consistently share information in a meaningful way to drive positive change and demonstrate the relationship between each department and its outcomes.
4. Efforts to contribute to personal development of individuals in each unit or department through added skillset, mentorship, coaching, and education.

A second session was held with unit representatives to identify a set of high-reliability behaviors at the unit level that would promote safe, high-quality, meaningful patient experiences. The representatives defined several essential behaviors, including:

- Promoting a just culture by encouraging all clinicians and staff to speak up for safety;
- Using multidisciplinary daily huddles to ensure situational awareness of high-risk topics like clinical concerns and environmental safety concerns;
- Using multidisciplinary and purposeful rounding;
- Ensuring clear and bidirectional communication;
- Learning from failures using data to drive quality improvement.

These activities have helped to strengthen the organizational values throughout the entire health system.

#### 3.4.2 Innovation units and the Bear’s Den

The Agile Institute has also formalized consultations by creating Innovation Units within each hospital of the health system. These units represent a variety of specialties and expertise (e.g., emergency medicine, telemetry, medical-surgical, etc.) and are leveraged to help discover and develop new solutions to improve care processes and outcomes. Some individuals within the Innovation Units have received training in Agile while others have not, but each is led and supported by the Agile Institute. The Innovation Units were chosen by nursing leadership based on demonstration of strong unit leadership, high performance, and consistent application of high-reliability behaviors. Once fully implemented, the Innovation Units can be called upon by clinicians and staff needing assistance innovating solutions, and they will help to implement sprints of small changes to reveal how best to achieve the desired change. The format and cadence of these sprints will be established through co-design sessions and regular virtual meetings with the team members and clinicians who submit the initial request. The Innovation Units will leverage Agile, including feedback loops and an agile mindset to rapidly pivot based on information gathered during the sprints.

The “Bear’s Den” is a collaborative space where team members, often in partnership with external companies, can develop and pitch new ideas related to improving care, often with a focus on technology solutions and those that enhance the patient experience. The Innovation Units have partnered with the Bear’s Den team to develop a streamlined “idea intake” form that can be submitted electronically from anywhere. The team is developing an evaluation tool that will help organize and prioritize ideas submitted for consideration based on criteria including cost, ease, impact, scalability, and strategic alignment. The Innovation Units have partnered with the Bear’s Den for idea intake and evaluation. They are a natural fit because they have a shared vision for excellence and integration upon successful implementation.

### 3.5 Organizational identity development (image building)

HMH understands that building a successful Agile Institute requires more than just sound methodology; it demands effective marketing, branding, and image building. These elements help to attract buy-in from key stakeholders, secure the necessary resources, and ultimately drive sustainable change. Image building efforts focused on both internal and external audiences. The goal of internal efforts was to reach key individuals and clearly communicate the Institute’s value proposition. This required identifying those key individuals and tailoring the message to resonate with them, whether they were clinicians, administrators, or staff. Storytelling was leveraged to spread the message of the Institute across a diverse set of internal communication channels such as internal newsletters, emails, presentations, workshops, as well as through external platforms like social media and conferences.

The Institute also developed a logo and built an internal website in preparation for creating an external-facing site. Various mediums were leveraged to promote the Institute externally. In addition to the website, social media, blogs, and YouTube allow for the Institute to reach a wide range of individuals across the healthcare landscape. When sharing information about the Institute, consistent visual images are used with the logo and specific imagery to reinforce brand recognition. On YouTube, the Institute broadcasts live interviews with individuals of interest, including members of the health system’s executive leadership. Additionally, individuals within the Institute disseminate research within the field of Implementation Science by submitting articles to relevant journals and speaking at conferences or summits.

The Institute also hosts various events for internal and external audiences. These include formal events to gather individuals from across the country to present and discuss ideas related to implementation science and Agile, and has also included a session held as part of the health system’s leadership event in the fall of 2024. The Institute also seeks to position itself as central for QI across the health system through integration in events like Quality Improvement Day and Culture Weeks. These solidify the Agile Institute as a leader in the promotion and execution of QI strategies that are effective in implementing sustainable change.

### 3.6 Measures of success and accomplishments to date

The success of the Agile Institute rests on its ability to accomplish the stated goals motivating its creation. Namely, whether the concepts and tools of Agile could be more quickly and consistently disseminated throughout the health system, and whether it allowed for more external collaborations with organizations who recognized the benefits allotted by Agile. To evaluate this, we can enumerate and quantify the specific accomplishments attributed to the Institute and those who have sought out its assistance. This is how the health system’s administration will determine whether the Institute is making a meaningful impact on the system.

**Figure 2:**
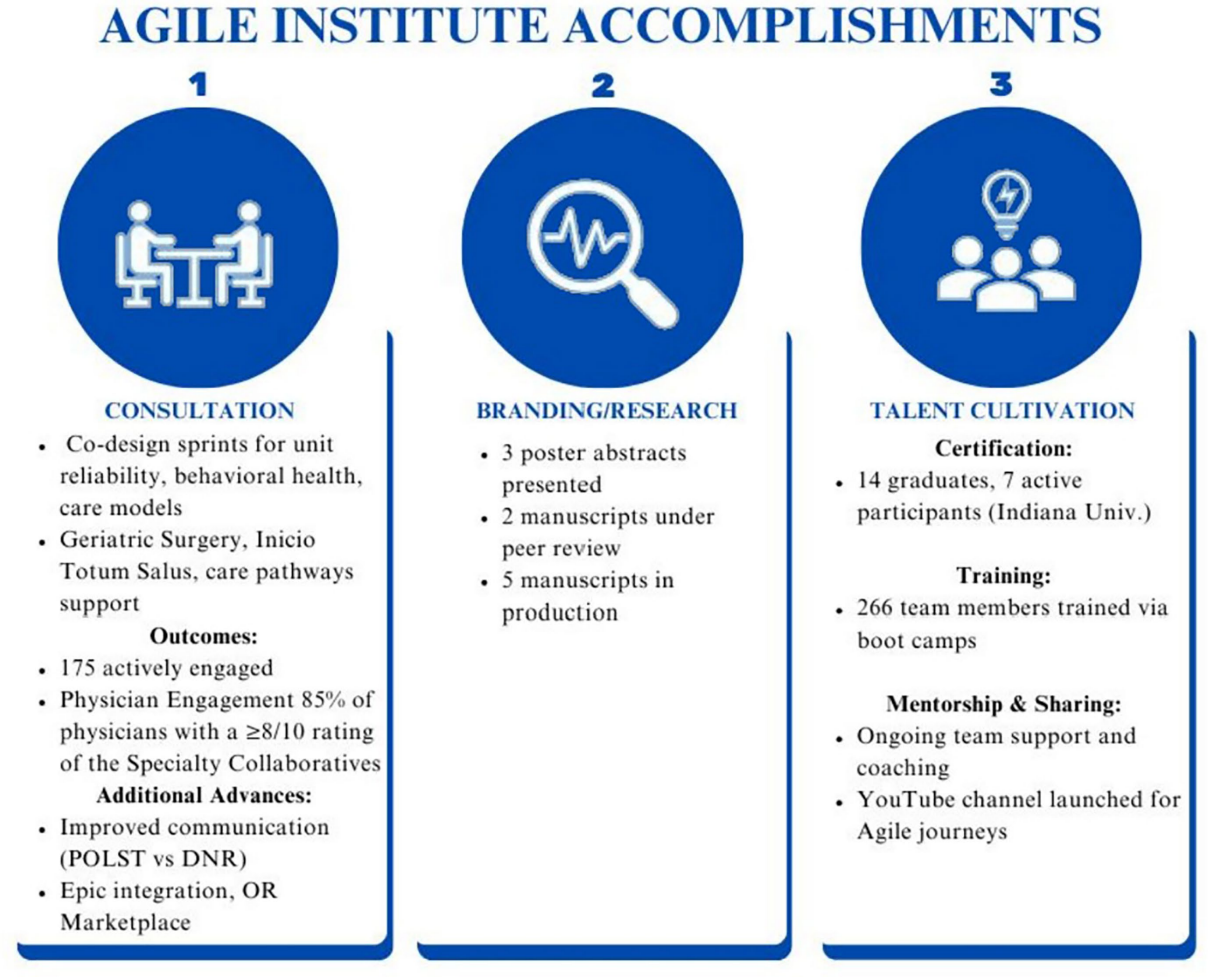
Accomplishments of the Agile Institute. Summary of key activities and outcomes across three domains: Consultation, Branding/Research, and Talent Cultivation. Consultation highlights collaborative initiatives and system Improvements; Branding/Research reflects scholarly dissemination efforts; Talent Cultivation includes certification, training, and mentorship. Abbreviations: POLST, Physician Orders for Life-Sustaining Treatment; DNR, Do Not Resuscitate.

In the past year, the Agile Institute has made significant strides in transforming healthcare delivery through strategic consultation, physician engagement, and talent development (Figure 2).

**Figure 3:**
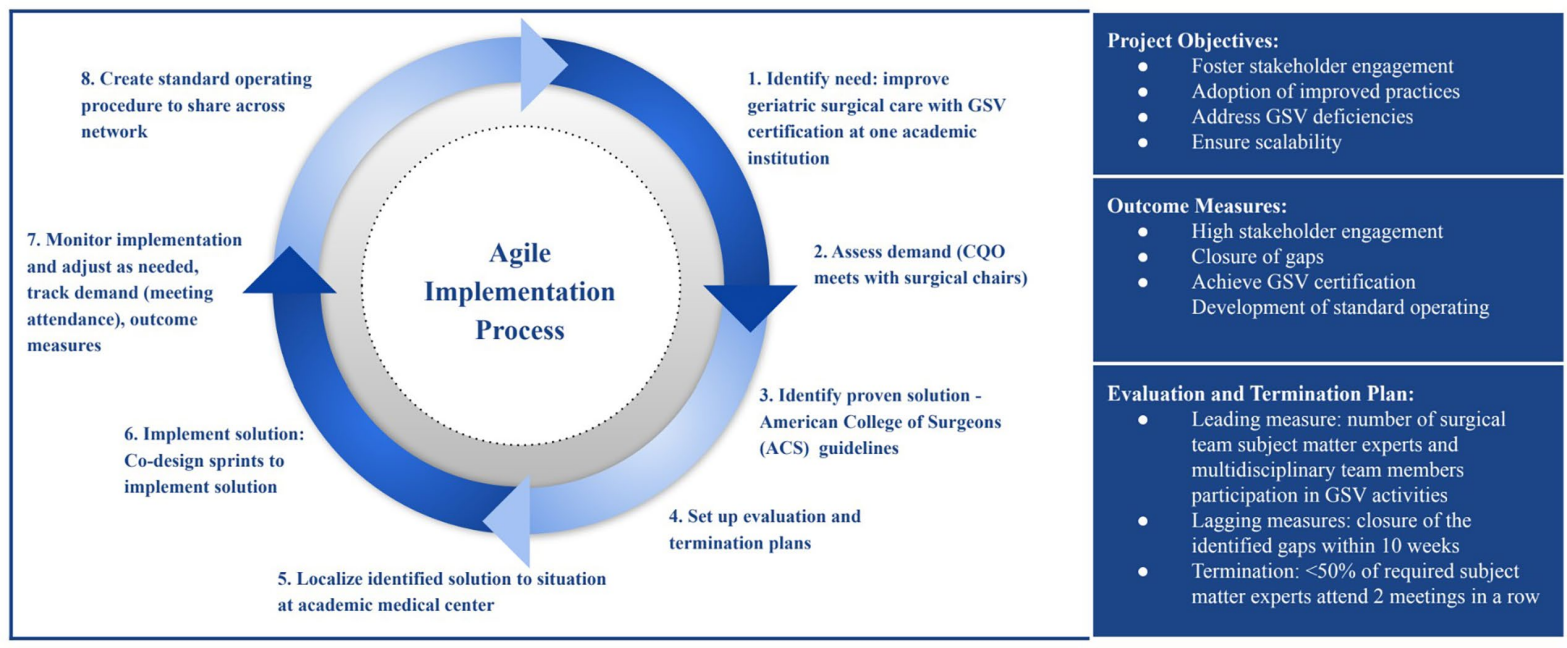
Agile implementation process for geriatric surgery verification. The Agile eight-step, iterative improvement process utilized for the rapid identification, implementation, and sustainment of interventions to address deficiencies and achieve geriatric surgery verification (GSV). CQO, chief quality officer.

The team facilitated multiple co-design sprints that addressed key initiatives such as unit-based reliability, innovation units, behavioral health transfer and care models, and the billing process for external partner services. Additionally, the team provided consultative support to critical projects, including a human resources culture initiative, the optimization of care pathways, and adherence to the geriatric surgery verification (GSV) program. Specifically, one of the academic medical centers within the system approached the Institute because of difficulty addressing multiple GSV program deficiencies, including Beers Criteria, delirium management, and care optimization. Together with representatives from the academic medical center, individuals at the Agile Institute developed a quality improvement plan to address the deficiencies that was rooted in Agile Science. This included confirming demand through conversations between the system’s Chief Quality Officer and the individual surgical chairs, selecting evidence-based solutions such as use of a GSV order set and delirium flowsheet, establishing regular meetings for co-design sprints where surgical staff and quality improvement staff could collaboratively develop improvement activities, and detailing an evaluation plan to track progress of the intervention. This plan included measures related to surgical team participation in the selected improvement activities, a specific timeline for measured improvement, and a “termination plan” that stated that if less than 50% of the required physicians and staff attended the regular meetings two times in a row, the intervention would be terminated and its structure reconsidered. While the surgical teams at the academic medical center were the drivers of the co-designed activities and the front-line changes to processes and systems in surgery to attempt to address the deficiencies, the individuals from the Agile Institute helped facilitate the regular meetings. They provided guidance for how best to measure, evaluate, and adjust the improvement activities to encourage change. In less than 10 weeks, all the identified GSV gaps had been successfully addressed, and a standardized operating procedure had been drafted that would allow the successful solutions to be implemented at other hospitals in the network if necessary (Figure 3).

As another example, the Institute launched the Specialty Collaborative to enhance collaboration across the health system, engaging 134 physicians across Surgery, Medicine, Behavioral Health, OB/GYN, and Pediatrics. Trained individuals from the Agile Institute led monthly meetings with clinician representatives from each hospital within the system. Collaborative discussions regarding improving care quality within each specialty fostered clinician involvement and input into quality improvement activities. During the meetings, agile-style sprints were held where clinician representatives identified care gaps, drafted clinical guideline recommendations, and priorities initiatives. These were systematically reviewed and integrated into the health system’s executive governance to ensure alignment with system goals. Success was measured, in part, by clinicians’ ratings of how effectively the meetings fostered open dialogue and addressed gaps in care. With 86% of participating physicians rating their experience as an 8-out-of-10 or higher, the collaboratives have fostered meaningful engagement and accelerated decision-making. These high ratings, in addition to consistently high attendance at the monthly meetings from each hospital’s assigned representatives, suggests that this Institute-initiated activity has been effective in securing clinician engagement.

The Agile Institute’s commitment to advancing healthcare innovation is reflected in its contributions to research and knowledge sharing. Over the past year, the team has produced three poster abstracts and has three publications currently in production, highlighting the impact of Agile in healthcare.

Talent cultivation remains a cornerstone of the Agile Institute’s mission. This year, five team members graduated from the Indiana University graduate certificate program and nine additional team members enrolled. Through monthly boot camps, the Institute has trained 136 team members, equipping them with the skills needed to drive Agile transformation. Ongoing mentorship and collaboration has further reinforced professional growth and development.

Through strategic consultation, innovative collaborations, and a commitment to continuous learning, the Agile Institute continues to drive meaningful transformation across the healthcare system. As the Institute moves forward, its focus remains on enhancing efficiency, fostering engagement, and leveraging technology to create a more agile and responsive healthcare environment.

## 4 Discussion

The creation of the Agile Institute at HMH emerged from a desire to incorporate Agile throughout the organization in order to facilitate rapid and sustainable improvement in governance, efficiency, cooperation, and the quality of care-delivery to enhance patient outcomes.

To approach and sustain behavioral change in a complex adaptive system such as the healthcare system, the Institute’s co-founders understood that the success of the Agile Institute would depend on various interventions at both the system level and the individual level. Securing system level leadership buy-in and clinician engagement was done through the use of frequent co-design sessions and consultation to build and sustain demand for change. The institute also provided training in Agile methodologies for individuals and offered guidance and support for specific interventions. This was done using Agile, creating minimally viable plans that can be localized to different settings, levering the use of sensors, and feedback loops with transparency in measurement and metrics related to the interventions (e.g., net promoter score and engagement rate for co-design sessions, termination plans for specific interventions, etc.).

The team modeled the agile mindset by applying the same concepts to their own approach to creating the Agile Institute, learning from failure when reaching a termination plan and pivoting. To achieve all of this, the co-founders of the Institute committed to dedicating time and space to build trust and strengthen relationships with one another, and to building a foundation of psychological safety. The result is that they have successfully created the Agile Institute and spread the use of Agile throughout the organization through training, consultation, and organizational identity development.

### 4.1 Practical implications

This case study highlights the effectiveness of Agile and illustrates its applicability across the entire care spectrum. HMH is a large system and is subject to regulatory, budgetary, and policy constraints while endeavoring to provide high-quality care to a variety of patient populations. Formalizing the mindset, tools, and techniques of Agile and encouraging their adoption and spread has led to higher clinical engagement, improved communication and cooperation among staff, personal and professional development of staff, and better patient experiences with care across the system.

### 4.2 Lessons learned for future applications

The results experienced by HMH through the development of the Agile Institute have implications for future endeavors and other organizations. For example, the team learned that the use of a minimally viable structure for the Institute allows them to effectively adapt to the needs of the network. This aligns with Agile methodologies and the agile mindset and stresses the importance of incorporating Agile into not only the activities but also the structure and administration of the Institute itself.

## 5 Conclusion

Given the challenges faced by today’s healthcare delivery systems and the challenges and barriers to QI and sustainable change that exist, the Agile Institute serves as an example of how organizations can leverage the concepts of Agile to transform themselves into cooperative, learning, and adaptive institutions. The activities promoted by the Agile Institute not only help to implement effective and sustainable improvement, but reach beyond QI efforts and help encourage culture change across the organization. Agile concepts related to psychological safety, co-design, and feedback loops are applicable for care delivery systems of all types, sizes, and make-ups.

## Data Availability

The raw data supporting the conclusions of this article will be made available by the authors, without undue reservation.
